# Spatial reach of the local field potential is frequency dependent

**DOI:** 10.1186/1471-2202-12-S1-P88

**Published:** 2011-07-18

**Authors:** Szymon Łęski, Henrik Lindén, Tom Tetzlaff, Klas H Pettersen, Gaute T Einevoll

**Affiliations:** 1 Department of Neurophysiology, Nencki Institute of Experimental Biology, Warsaw, 02-093, Poland; 2 Department of Mathematical Sciences and Technology, Norwegian University of Life Sciences, Ås, 1432, Norway

## 

The local field potential (LFP), the low-frequency part of the extracellularly recorded electric potential, is a signal commonly used to study the activity of neural populations. However, it is not clear how *local* the ‘local’ field potential in fact is, i.e., how large the populations are which contribute to the signal at a given point [1]. The spatial range of LFP will naturally depend on the spatial range of correlations in the neuronal dynamics, which may be different in different frequency bands. However, we expect that also the inherent low-pass filtering in the neuronal cables, which stems from the properties of the cable equation [2], will contribute to the frequency dependence of the spatial range of the LFP.

**Figure 1:**
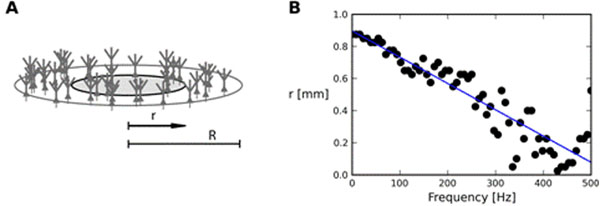
A. Model setup, *r* (reach) and *R* defined in text. B. The reach *r* as a function of LFP frequency for a population of layer 5 pyramidal neurons stimulated basally. The input correlation is 10% (each pair of neurons shares on average 10% of synaptic currents), LFP measured at the soma level.

To investigate how dendritic filtering affects the spatial range of different frequency components of the LFP we simulated a population of morphologically reconstructed neurons homogeneously distributed within a disc of radius *R* = 1mm (Figure 1A). The cells were driven by noise input of varying degree of correlation. We calculated the LFP at the center of the disc. We defined the *reach* of the LFP as radius *r* <*R* such that the cells located closer than *r* account for 95% of the power of the LFP. We found that for some combinations of neuron morphology, stimulation pattern, and degree of correlation, the reach of the high-frequency (> 100 Hz) components can be even four times smaller than the reach of the low-frequency (< 50 Hz) components (Figure 1B).

The explanation of the effect is the following: consider a neuron stimulated with a sinusoidal input current (frequency *f*) at one point in the dendritic tree. The distribution of the return currents depends on *f*, with typically more local return currents for large *f*, see [*3*]. Therefore the ‘critical distance’, where the dipole approximation of the LFP becomes valid, decreases with *f*. We derived a simplified population model that accounts for the frequency dependence of the ‘critical distance’. The model can be studied analytically and explains the frequency dependence of the LFP reach for both uncorrelated and correlated synaptic input.
